# Differences in Trajectories and Predictive Factors of Cognition over Time in a Sample of Cognitively Healthy Adults, in Zaragoza, Spain

**DOI:** 10.3390/ijerph18137092

**Published:** 2021-07-02

**Authors:** Elena Lobo, Patricia Gracia-García, Antonio Lobo, Pedro Saz, Concepción De-la-Cámara

**Affiliations:** 1 Department of Preventive Medicine and Public Health, Universidad de Zaragoza, 50009 Zaragoza, Spain; elobo@unizar.es; 2 Instituto de Investigación Sanitaria de Aragón (IIS Aragón), 50009 Zaragoza, Spain; pgraciag@salud.aragon.es (P.G.-G.); psaz@unizar.es (P.S.); conchidlc@hotmail.com (C.D.-l.-C.); 3 Centro de Investigación Biomédica en Red de Salud Mental (CIBERSAM), Ministry of Science and Innovation, 28029 Madrid, Spain; 4 Psychiatry Service, Hospital Universitario Miguel Servet, 50009 Zaragoza, Spain; 5 Department of Medicine and Psychiatry, Universidad de Zaragoza, 50009 Zaragoza, Spain; 6 Psychiatry Service, Hospital Clínico Universitario, 50009 Zaragoza, Spain

**Keywords:** aging, cognition, memory trajectories, ZARADEMP Study

## Abstract

Great inter-individual variability has been reported in the maintenance of cognitive function in aging. We examined this heterogeneity by modeling cognitive trajectories in a population-based longitudinal study of adults aged 55+ years. We hypothesized that (1) distinct classes of cognitive trajectories would be found, and (2) between-class differences in associated factors would be observed. The sample comprised 2403 cognitively healthy individuals from the Zaragoza Dementia and Depression (ZARADEMP) project, who had at least three measurements of the Mini-Mental State Examination (MMSE) in a 12-year follow-up. Longitudinal changes in cognitive functioning were modeled using growth mixture models (GMM) in the data. The best-fitting age-adjusted model showed 3 distinct trajectories, with 1-high-to-moderate (21.2% of participants), 2-moderate-stable (67.5%) and, 3-low-and-declining (9.9%) cognitive function over time, respectively. Compared with the reference 2-trajectory, the association of education and depression was significantly different in trajectories 1 and 3. Instrumental activities of daily living (iADLs) were only associated with the declining trajectory. This suggests that intervention strategies should be tailored specifically to individuals with different trajectories of cognitive aging, and intervention strategies designed to maintain cognitive function might be different from those to prevent decline. A stable cognitive performance (‘successful cognitive aging’) rather than a mild decline, might be more ‘normal’ than generally expected.

## 1. Introduction

The interest of public health in cognitive health in old age has grown in the last decade worldwide as cognitive function is considered a major determinant of healthy aging [1]. There is abundant scientific literature about the risk and protective factors of cognitive impairment and dementia [2,3,4]. However, more studies are required about the maintenance of cognitive abilities and their determinants, and disease-focused research seems insufficient to identify such factors [5].

Cognitive aging refers to the process of cognitive decline that occurs as people get older. It is often argued that age-related changes in the abilities of reasoning, memory, and processing speed may arise during adulthood and progress into the elderly. Nevertheless, there is not a common patron of deterioration and some individuals may not ever decline, maintaining their abilities in a good condition [6]. This could be because chronological age may be a poor proxy for biological age [7].

Importantly, evidence suggests that the broad spectrum of changes in cognition includes a large inter-individual and intra-individual variability across the later life span. An individual’s performance on measures of ability may change across evaluation moments [8] so that longitudinal repeated cognitive assessments are better than a cross-sectional measure to capture the natural process of cognitive aging over time [9]. The population heterogeneity observed in some previous studies increases the challenges to understand the process of cognitive aging itself as well as the influence of factors causally associated with cognitive aging. The extensive research literature aiming to identify risk and protective factors has focused on the comparison of those impaired or demented with the non-impaired group assuming that both groups are homogeneous. However, a different research approach may be required, since there is an emerging body of knowledge suggesting that there may be more than two patterns of change in later-life cognition [10,11,12,13,14].

Furthermore, factors associated with the different trajectories may not be the same. As an example, Terrera et al. showed that education was only protective for the subjects with good performance in cognition, but did not affect the other groups [14]. Recently, McFall et al. observed that distinct modifiable factors were predictive of class membership, suggesting that targets for promoting healthy memory aging may differ from targets for delaying decline [15]. These findings also remark that the “successful cognitive aging” challenge remains under debate, and the factors that distinguish high cognitive performance from ‘normal aging’ require new empirical studies.

The methodological approach most used in previous studies to explore the patterns of cognitive capacity in older adults over time is the Growth Mixture Models (GMM). This technique allows for detection when a group of individuals changes on the cognitive measure. Since this method takes into account both baseline and longitudinal changes, it also permits to see if there are individual differences in the rates of change among group members [16].

A recent review of reports on this topic observed intriguing between-study differences, although they used similar procedures for the analysis [17]. The number of classes identified varied from two to six groups and the proportion of individuals falling into each of these classes varied considerably throughout studies. This could be explained partly by some differences in the outcome measures but also by distinctions in the samples used. With regards to the factors analyzed as predictors of class membership, Wu et al. synthesized that those being younger, female, with higher education, better health conditions, and without a genetic risk factor were more likely to be in a higher-performing group, however, some opposing findings and inconsistencies have also been seen in the literature and might be overcome by designing new studies with improved methodologies. While age has not predicted a worse cognitive pattern in all the studies, we consider it is an important confounding factor to be controlled for when modeling the cognitive patterns. Referring to the potentially modifiable factors, depression has been suggested in some previous studies to be associated with cognitive decline trajectories [9,10,11,15,18,19]. However, these studies have some limitations, since depression was assessed with screening questionnaires, and not with standardized interviews specifically designed to document clinically significant, treatable depression in the elderly. Furthermore, anxiety has not been considered in those studies on cognitive aging trajectories. However, anxiety merits consideration, since recent reports have shown its association with incident cognitive impairment and dementia [20].

Except for one study in Spain analyzing trajectories of verbal memory [21] and the PAQID [22] and the INSIGHT-PreAD [23] studies in France, there are no data from Southern Europe or in other Mediterranean countries modeling cognitive trajectories over time. North-Western vs. Southern European cities’ geographical differences in the incidence of dementia have been reported previously [24], as well as lower incidences of dementia in Zaragoza versus other European and non-European cities [25]. Therefore, the study of cognitive trajectories in Zaragoza might have additional interest to eventually explore the possibility that the cognitive pattern has some differences concerning studies reported in other socio-cultural regions.

The present paper aims to build on previous research in several ways. The objective was to identify patterns of cognitive change in a 12-year follow-up in a sample of healthy cognitive adults of 55+ years in Spain, including age at baseline for the data modeling. We hypothesized that distinct cognitive trajectories would be found. Concerning predictive factors, between-trajectory differences might be expected, and we hypothesized that education would be a protective and a risk factor for cognition maintenance and decline, respectively; we also hypothesized that both depression and anxiety would predict trajectories of cognitive decline.

## 2. Materials and Methods

### 2.1. Sample and Procedure

This study is a secondary analysis using data from the ZARADEMP project [25,26], a longitudinal population-based study of dementia and depression in adults aged 55 years or older, conducted in Zaragoza, Spain. The general methods have been previously reported [26].

A random, representative sample of the city (700,000 inhabitants) was drawn from the individuals registered in the Zaragoza official census lists. It included institutionalized individuals and was stratified with proportional allocation by age and sex. The refusal rate was 20.5%, and 4803 individuals were ultimately interviewed at baseline (wave I, 1994). Individuals with dementia as well as those with “subsyndromal” dementia at baseline, according to the Geriatric Mental State (GMS), with its cognitive section and its Automated Geriatric Examination for Computer Assisted Taxonomy package (AGECAT) criteria [27], were excluded for the follow-up waves.

In summary, the procedure included a two-phase case finding. In phase I, well-trained and regularly supervised lay-interviewers administered the ZARADEMP interview at the participant’s home or place of residence. In phase II, the trained research psychiatrist reassessed participants to confirm the suspected clinical diagnosis of dementia, as well as the presence of depression or anxiety identified in phase I. The validity of this approach has been established [27]. A similar procedure was implemented in the second, third, and fourth waves (2.5, 4.5, and 12 years later, respectively), in which interviewers were not aware of the results of the baseline interview described beforehand.

The Helsinki convention principles of written informed consent, privacy, and confidentiality have been maintained throughout the Project, and the Ethics Committee of the University of Zaragoza and the Fondo de Investigación Sanitaria (FIS) approved the study (CP16/2012, 19 September 2012), according to Spanish Law.

### 2.2. Assessment of Cognitive Function

The diagnostic tools and questionnaires used in the ZARADEMP were validated, Spanish versions of international instruments. Besides GMS and AGECAT, previously mentioned, for this study, cognitive status was assessed using the Mini-Mental State Examination MMSE [28]. This tool assesses cognition measuring orientation in time and space, memory, attention, calculation, language, and visuoperception. The Spanish version of the test [29] was administered according to standardized procedures, and the total scores range from 0 to 30, with higher scores representing higher cognitive functioning.

### 2.3. Other Measurements

At the baseline assessment, participants provided information on factors potentially associated with cognitive decline identified and used in the literature, including the publications on cognitive trajectories and our previous studies conducted on the ZARADEMP cohort. Of interest for inclusion as covariates in this study are the following: age, education (“Illiterate”: unable to read and write, or with less than 2 years of formal education; “Primary”: primary studies, up to 8 years of formal education; and “Medium/High”: secondary education or above with more than 9 years of formal education) and marital status (single, including separated, divorced, monk/nun; living with a couple; widowed), health behaviors such as tobacco use and alcohol intake, and the medical diagnosis of diabetes and hypertension. Dependency in Basic and Instrumental Activities of Daily Living, were also ascertained. These variables were coded based on the information obtained from the History and Aetiology Schedule (HAS), disability scales (Katz’s Index) for basic activities of daily living (bADLs) and Lawton and Brody scale for iADLs, and the European Studies of Dementia (EURODEM) Risk Factors Questionnaire for the assessment of medical conditions [25]. Depression and anxiety syndromes were assessed utilizing the GMS interview and the respective sections in the AGECAT computer system. In phase II, the trained research psychiatrists diagnosed the presence of depression and/or anxiety syndromes according to the standard AGECAT criteria, in participants scoring 3, 4, or 5 on the 0–5 scales.

### 2.4. Data Analysis

Longitudinal changes in cognitive functioning were modeled using growth mixture models (GMM) to the data [30,31]. This approach is capable of detecting not only whether a group of individuals changes on some outcome measure, but whether there are individual differences in the rates of change among group members [32].

This technique estimates individually varying trajectories of change for the outcome measure, acknowledging the heterogeneity in change over time but also allowing for the identification of subpopulations or groups of individuals with similar patterns of change. It also allows to model data from participants with different baseline ages and numbers of time points [33].

The model classifies individuals into clusters with similar trajectories according to their longitudinal data, assuming that individual differences in trajectories can be summarized by a finite set of different polynomial functions for age or time [34,35].

Using lcmm package of R software, linear and non-linear link functions, specifically Beta cumulative distribution functions and splines, were used to estimate the best GMM. The time of the MMSE measurement, age at baseline, and the interaction between them were all incorporated in the models; including random effects in the time covariate.

All the individuals in the data set with at least three measurements of MMSE were included in the analyses, therefore individuals with one MMSE measure missing data were not removed from the analysis.

Analyses were carried out to fit latent-class solutions to the data. The best solution was selected after examining fit indices such as the Akaike Information Criterion (AIC), Bayesian Information Criterion (BIC), the sample-size-adjusted BIC (SABIC), and considering the solution’s interpretability and parsimony. Lower criterion values indicate better model fit. Additionally, based on Jung et al.’s criteria, a proportion of each class (no less than 1%) was considered [36].

Baseline characteristics were expressed as mean and standard deviation (SD) for continuous variables and proportions for categorical variables. Chi-squared test (for categorical variables) and one-way analysis of variance (ANOVA) with the Snedecor-F test (for continuous variables) were used to compare the differences among the trajectory groups. Multinomial logistic regression (MLR), with the most prevalent class as the referent [12], was applied to identify baseline variables, including sociodemographic, physical, mental, and lifestyle predictors of cognitive function trajectory class. 

All analyses were implemented using R software (R Foundation for Statistical Computing, Vienna, Austria) (http://www.r-project.org, accessed on: 21 March 2021).

## 3. Results

A total of 2403 subjects of the ZARADEMP study had MMSE measures in at least three waves and was included in the analyses. At baseline, the percentage of women was 55.9% and the average age was 70.3 years old. The majority of the sample had primary or less education (80.1%) and were married or were a couple (67.8%). Among participants, hypertension and diabetes were present in 67% and 11.7%, respectively. Depression syndromes were observed in 15.8% of the subjects and anxiety syndromes in 4% of them. Some participants were dependent on basic and instrumental ADLs (4.2% and 7.6%, respectively). Referring to alcohol intake, 60.5% of the sample never drunk, while 24% used to drink regularly, 4.8% occasionally and 10.6% were ex-drinkers. Most of them were non-smokers (64.3%) and, 13.8 and 21.9% were smokers and ex-smokers, respectively.

Globally, the MMSE performance for the whole sample showed a declining trend in cognitive function over time. At baseline (Wave 1), the average score of MMSE was 27.6 (2.2) out of 30. Across the 4 waves over 12 years, the average scores remained above a score of 26 points. The MMSE means in Waves 2 to 4 were 27.5 (2.6), 26.8 (3.9), and, 26.7 (4.7), respectively.

To capture inter-individual variability in the cognitive performance during follow-up, a total of 12 series of GMM were fit to the data. Models with more than 3 classes were discarded due to a very low proportion of individuals in some of the estimated classes (<1%). After careful examination of the statistical parameters, the best fitting age-adjusted models of the cognitive trajectories were a 2-class and a 3-class growth model. The rationale for the selection between them was based on the fact that, while the BIC was somewhat lower for the 2-class model (33,308.49) than the 3-class model (33,311.26), the AIC, SABIC and negative Log-likelihood for the 3-class model (33,224.50, 33,263.61, and −16,597.25, respectively) were lower than the 2-class model (33,244.86, 33,273.54, and −16,611.43, respectively) (Table 1).

**Figure 1:**
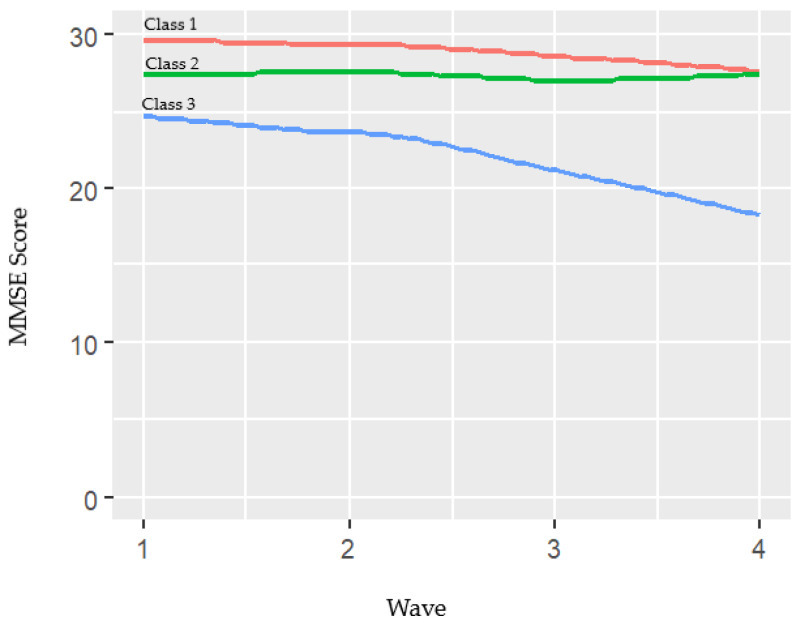
Growth mixture model estimated means of MMSE (cognitive trajectories) over 12 years of follow-up: ZARADEMP Study.

Figure 1 illustrates the trajectories of the three classes and Table 2 shows details of the MMSE mean scores for each class and wave. Participants in Class 1 (21.2%) began with a high MMSE mean at baseline (29.6; SD = 0.6) and declined slightly and gradually at follow-up until 27.6 points at Wave 4. Participants in Class 2 were the most numerous (68.9%) and began with a moderate MMSE mean score (27.4; SD = 1.7) which remained stable over time until wave 4, with the MMSE rating equal or above 27 points during the entire follow-up. Subjects in Class 3 (9.9%) began with a moderate score in MMSE (24.6; SD = 3.2) and declined slowly from baseline to wave 2 (23.6) and, then, more rapidly to waves 3 (21.1) and 4 (18.2). The posterior probabilities for Class 1, 2, and 3 were 74.8%, 73.8%, and 69.8%, respectively (Table 2).

The three trajectory groups significantly varied in sociodemographic characteristics, lifestyles, and health factors (Table 3). At baseline, compared with participants in Classes 2 and 3, individuals in Class 1 were more likely men, single or living with a couple. They also had a higher degree of education and were more likely habitual alcohol drinkers and/or smokers; and, less frequently had depression, anxiety, and/or dependency on instrumental ADLs.

Compared with participants in Classes 1 and 2, individuals in the more declining group (Class 3) were more frequently women, widowed, illiterate and with primary education, never drinkers or smokers, and the percentage of HTA, depression, anxiety and dependency on instrumental ADLs was higher among them.

Table 4 shows the results of the multinomial logistic regression with the baseline characteristics predicting class membership. Similar results were observed in the bivariate analysis. After controlling for potential confounders, compared with Class 2, individuals with a higher level of education were more likely to appear in Class 1 and illiterates in Class 3, and depressed subjects were more likely to be in Class 3, and individuals without depression in Class 1. Compared with class 2, those dependent on instrumental ADLs were more likely to belong to Class 3. No association was found between alcohol or smoke habits, neither with hypertension or anxiety.

## 4. Discussion

In support of the first hypothesis, this study has found three distinct age-adjusted trajectories of cognitive aging in 55+ years old adults over a follow-up of 12 years. In partial support of the second hypothesis, the study has found between-group differences in the factors associated. Both education and depression were predictors of class membership for all the trajectories 1–3 (high to moderate, moderate-stable, and low-and-declining cognitive function over time, respectively). Moreover, both depression and instrumental dependency in ADLs predicted the pattern of low-and-declining cognitive function.

In coincidence with traditional knowledge, the MMSE mean score for the total sample declined over the follow-up. However, the GMM methodology, which allows a more detailed analysis, has documented that this trend was not consistent when looking at each identified class. We observed that individuals in trajectory class 3 had a low cognitive performance at baseline and pronounced cognitive decline, and individuals with higher cognitive performance at baseline were in the two other classes: one with the highest MMSE mean score at baseline and with a slight decline over time (class 1) and the other, which represents the majority of the sample (class 2), starting with moderate MMSE mean score and keeping a very stable pattern throughout the follow-up.

Our results are consistent with previous findings in the number of classes identified [17]. It might be argued that the trajectories of classes 1 (high-to-moderate) and 2 (moderate-stable) almost reach the same point at the end of follow-up (27.6 and 27.4, respectively). Nevertheless, the MMSE mean scores of both groups did not overlap at any moment and a longer follow-up might show clearer between-trajectory differences. In some previous studies with long follow-up it has been shown that in similar circumstances, in trajectories that flew along with similar MMSE scores, the outcome was different. For example, higher mortality was observed in trajectories of higher decline [10].

This study also confirms that in order to capture the natural process of cognitive aging, longitudinal repeated cognitive assessments, as collected here, are better than a cross-sectional measure [9]. For example, it was unexpected, and remarkable, that compared with subjects in class 1 (high-to-moderate), whose cognitive performance declined in the study period, participants in class 2 (moderate-stable) had lower mean MMSE scores at baseline but their cognitive performance remained stable and at the end of follow-up was very close to the mean MMSE score in individuals in class 1 (27.4 vs. 27.6 respectively). At this point, we do not have a good explanation for this finding. However, since participants in Class 2 (moderate-stable) were the most numerous (more than two thirds), should this finding be replicated in future studies, it may suggest that a stable cognitive performance (‘successful cognitive aging’), rather than a mild decline, is ‘normal’ in the general population.

Another important finding of the present study refers to the predictors of class membership. Regarding education, Terrera et al., observed a protective effect only for the good-performers [14]. However, compared with the reference 2-trajectory (moderate-stable), we have seen that the academic level has a link both with the 1-‘high-to-moderate’ and the 3-‘low-and-declining’ cognitive function trajectories. The highest level of education was linked with the highest probability of belonging to trajectory-1 and, on the contrary, the lowest level of education was associated with the declining trajectory. Regarding the important role of education on cognitive performance, we have participated in a large, cross-national study showing that every additional year of education was associated with a rate of decline slightly slower for the MMSE across samples from diverse ethno-cultural groups and geographical regions, although associations varied across cohorts [37]. This supports the importance of developing policies to improve education early in life, particularly in populations with high rates of illiteracy or low education, to promote the healthiest cognitive aging. On the other hand, the literature suggests that keeping an active cognitive life has positive effects [38], although it remains to be documented to what extent specific educational measures in adult age may still be considered preventive of cognitive decline. Besides, any global prevention strategy may need to consider ethno-regional differences [39].

Depression is the other factor that, compared with the reference 2-trajectory, has links with both, the ‘high-to-moderate’ and the ‘low-and-declining’ cognitive function trajectories. The confirmation that it was associated with the declining category and, on the contrary, the absence of depression was related to trajectory-1 with high cognitive performance at baseline is remarkable. These results are generally in accordance with the previous literature. Firstly, depression has been considered a risk factor for cognitive decline and dementia [40,41,42]. Secondly, depression may exacerbate cognitive difficulties and disability, especially in older adults [43]. To note in this study is the fact that, contrary to most previous reports in the literature, which assessed depression using self-report symptomatic scales, we used the GMS-AGECAT instruments, considered to be valid methods to detect clinically significant depression in elder people [44].

Anxiety was found to be predictor of class membership in the univariate analysis, the proportion of participants with this syndrome being higher in the category with lower cognitive performance (class 3: low-and-declining); but was no longer considered an independent predictor in the multivariate analysis. We have previously shown that anxiety as a clinical diagnostic category increases the risk of AD, although the association coefficient was stronger in cases of depression [45]. A possible reason for the discrepant results may be related to the different outcome measures and statistical analyses. In this particular study, we assessed anxiety syndromes, and have subjected the anxiety variable to a stringent statistical test since anxiety commonly accompanies depression in the same individuals [46] and depression was controlled for in the multivariate analysis. Since we suspected collinearity between depression and anxiety syndromes, this possibility was tested and our prediction was confirmed.

We found that dependency in instrumental ADLs was a predictor of class 3 (low-and-declining) membership, the category with clearer cognitive deterioration. Our results are consistent with those of Farias et al. [47]. These authors found that among participants initially characterized as cognitively normal, those who showed a cognitive decline over the follow-up period already demonstrated greater functional impairment as compared with those who remained cognitively normal. Some previous studies, including a relevant multi-center report in a mild AD sample, have also documented that cognitive decline precedes functional impairment [48]. Nevertheless, our results suggest that an inverse relationship may also be found.

Some features in this study were associated with all the trajectory classes. Specifically, compared with the reference class (class 2, moderate-stable), a high educational level was associated with class 1 (the ones with better cognitive performance: high-to-moderate), and the opposite, low educational level was associated with the low cognitive level class. Similarly, compared with the reference class 2, depression was associated with cognitive deterioration in individuals in class 3 (low-and-declining), and the opposite, absence of depression was associated in individuals in class 1. However, the association of other feature, specifically, dependence in iADLs only predicted belonging to one of the categories, class 3 (low-and-declining) when compared to the reference class 2; but the opposite was not true, since the association of independence in iADLs did not predict belonging to class 1. Similar observations have been reported before [15], and this might have important implications for prevention and healthy cognition promotion, as looking for mechanisms of cognitive maintenance and resilience might not simply be to look for the opposite of those identified as risk factors for deterioration [17]. 

Our results support the trajectory model of research in cognitive aging and reinforce the importance of identifying predictors of cognitive change in “cognitively normal” subjects for early identification of cognitive impairment and possible/probable dementia [49]. To some extent, the findings on predictors of cognitive decline reinforce previous knowledge and public health measures to prevent cognitive impairment and dementia. However, the finding that cognitive performance remained stable throughout a long follow-up period in a high proportion of individuals in this sample should be emphasized. The finding suggests that this sector of the population with stable cognitive performance should be thoroughly investigated to identify potential protective factors to eventually implement adequate public health policies.

The methodology used in this study has other strengths. Unlike some previous studies [17], this one used a rigorous screening procedure to recruit individuals who were cognitively healthy at baseline. It is relevant to note that even excluding cases and subcases of dementia at baseline, it was possible to identify among cognitively healthy individuals distinct trajectories of cognition in the follow-up. 

Moreover, to our knowledge, this is the first report modeling trajectories incorporating clinically significant syndromes of both depression and anxiety as potential predictors of class membership. Finally, differently from most previous studies, age at baseline has been considered an important potential confounder in the identification of patterns and was included and controlled for in the trajectory modeling.

This study has several limitations. First, the stringent exclusion of cases but also subcases of dementia does not allow the observation of trajectories in individuals with non-severe cognitive deficits. Second, the baseline modifiable factors studied, such as comorbidities or psychosocial conditions, might have changed during follow-up, a subject that merits future lines for research. Third, we cannot discard the influence of factors non-controlled in this study, such as frailty, social engagement, or APOEe4. And fourth, a mediation statistical model would allow to better analyze which factors may be cause or effect of belonging to one class or another.

## 5. Conclusions

The findings of the present study confirm that controlling for age at baseline, there are distinct heterogeneous trajectory patterns in cognitive function among a general population of cognitively healthy adults of 55+ years. Specifically, we have identified 3 groups, with 1-high-to-moderate, 2-moderate-stable, and 3-low-and-declining cognitive function, respectively. Since more than two-thirds of participants were included in the 2-trajectory, the suggestion is that a stable cognitive performance (‘successful cognitive aging’) rather than a mild decline, might be more ‘normal’ than generally expected.

Furthermore, our results suggest that some factors predicting class membership are different for each trajectory. Compared with the reference 2-trajectory, the association of education and depression was significantly different in trajectories 1 and 3. A higher level of education and not having depression predicted being in the trajectory group with higher cognitive performance at baseline, but a lower level of education and depression predicted belonging to the class with clear cognitive decline. Moreover, dependency in iADLs was predictor only for the declining group.

This remarks the importance of taking into account the heterogeneity in cognitive aging when looking for both, predictors of decline and predictors of maintenance of cognitive performance, which may be different and not just simply opposites.

## Figures and Tables

**Table 1:** Fit statistics for the grow mixture models of cognitive trajectories from ZARADEMP Study.

Total observations	8406	
*N*	2403	
nº classes	2	3
AIC ^1^	33,244.86	33,224.5
BIC ^2^	33,308.49	33,311.26
SABIC ^3^	33,273.54	33,263.61
maximum log-likelihood	−16,611.43	−16,597.25
Entropy	0.55	0.45

^1^ AIC: Akaike information criterion. ^2^ BIC: Bayesian information criterion. ^3^ SABIC: Sample-size adjusted BIC.

**Table 2:** Characteristics of the Trajectories Identified (Classes 1–3) and changes in cognitive function over 12 years: ZARADEMP Study.

Class	*n* (%)	Posterior Probability (%)	MMSE Scores				
				Wave 1 (Baseline)	Wave 2	Wave 3	Wave 4
1	510 (21.2)	74.8	Mean (SD)	29.6 (0.6)	29.3 (0.9)	28.6 (1.7)	27.6 (3.9)
			Min-max	27–30	25–30	18–30	0–30
2	1656 (68.9)	73.8	Mean (SD)	27.4 (1.7)	27.5 (1.9)	27.0 (2.8)	27.4 (3.0)
			Min-max	18–30	18–30	0–30	3–30
3	237 (9.9)	69.8	Mean (SD)	24.6 (3.2)	23.6 (4.5)	21.1 (6.9)	18.2 (8.1)
			Min-max	0–29	0–30	0–29	0–28

**Table 3:** Baseline characteristics of individuals in Classes 1–3.

	Class 1 *N* = 510 (21.2%)	Class 2 *N* = 1656 (68.9%)	Class 3 *N* = 237 (9.9%)	*p*-Value
Age	69.6 (7.6)	70.4 (8.1)	70.4 (7.4)	0.107
Men	259 (50.8)	727 (43.9)	75 (31.7)	<0.001
Education				
Illiterate	4 (0.8)	105 (6.3)	42 (17.7)	<0.001
Primary	286 (56.1)	1301 (78.6)	186 (78.5)	
Medium/High	219 (42.9)	239 (14.4)	8 (3.4)	
Missing	1 (0.2)	11 (0.7)	1 (0.4)	
Marital Status				
Single ^1^	60 (11.8)	171 (10.3)	20 (8.4)	
Couple	370 (72.6)	1105 (66.7)	154 (65.0)	0.003
Widowed	79 (15.5)	376 (22.7)	62 (26.3)	
Missing	1 (0.2)	4 (0.2)	1 (0.4)	
Hypertension	338 (66.3)	1095 (66.1)	177 (74.7)	0.032
Missing	0 (0.0)	3 (0.2)	0 (0.0)	
Diabetes	51 (10.0)	193 (11.7)	38 (16.0)	0.054
Missing	2 (0.4)	13 (0.8)	2 (0.8)	
Depression	49 (9.6)	271 (16.4)	59 (25.0)	<0.001
Missing	2 (0.4)	53 (3.2)	27 (11.4)	
Anxiety	13 (2.6)	62 (3.7)	20 (8.4)	<0.001
iADLs ^2^	24 (4.7)	123 (7.4)	35 (14.8)	<0.001
Missing	0 (0.0)	1 (0.1)	3 (1.3)	
bADLs ^3^	19 (3.7)	71 (4.3)	12 (5.1)	0.688
Missing	1 (0.2)	2 (0.1)	1 (0.4)	
Alcohol				
Ex-drinker	45 (8.8)	186 (11.2)	24 (10.1)	0.002
Habitual	140 (27.5)	396 (23.9)	41 (17.3)	
Never	289 (56.7)	998 (60.3)	166 (70.0)	
Ocassional	35 (6.9)	75 (4.5)	6 (2.5)	
Missing	1 (0.2)	1 (0.1)	0 (0.0)	
Smoking status				
Ex-smoker	125 (24.5)	361 (21.8)	39 (16.5)	<0.001
Non-smoker	292 (57.3)	1079 (65.2)	175 (73.8)	
Smoker	92 (18.0)	216 (13.0)	23 (9.7)	
Missing	1 (0.2%)	0 (0.0)	0 (0.0)	

^1^ Single: includes separated, monk/nun. ^2^ Dependency in instrumental ADLs. ^3^ Dependency in basic ADLs.

**Table 4:** Multinomial Logistic Regression of Predictors of Class membership.

	Class 1 ^1^			Class 3 ^1^		
	OR	95% CI	*p*-Value	OR	95% CI	*p*-Value
Men	0.93	0.72–1.21	0.657	0.72	0.47, 1.08	0.182
Education (ref. Illiterate)						
Primary	5.12	2.19–11.96	0.002	0.36	0.25–0.52	<0.001
Higher	20.33	8.62–47.95	<0.001	0.11	0.05–0.22	<0.001
Marital Status (ref. Coupled)						
Single ^2^	0.99	0.74–1.32	0.938	0.77	0.49–1.20	0.332
Widowed	0.82	0.63–1.05	0.187	0.81	0.59–1.10	0.259
Hypertension	1.10	0.91–1.33	0.396	1.38	1.04–1.84	0.061
Depression	0.62	0.46–0.83	0.007	1.50	1.11–2.02	0.027
Anxiety	0.86	0.50–1.48	0.645	1.60	0.98–2.62	0.114
iADLs Dependency ^3^	0.79	0.52–1.18	0.329	1.85	1.26–2.70	0.008
Alcohol (ref. Never)						
Ex-drinker	0.81	0.58–1.32	0.305	0.94	0.59–1.49	0.830
Habitual	0.98	0.77–1.24	0.878	0.92	0.63–1.33	0.704
Ocassional	1.10	0.75–1.63	0.679	0.68	0.31–1.52	0.434
Smoking Status (ref. Non-smoker)						
Ex-smoker	0.99	0.76–1.30	0.967	1.06	0.69–1.65	0.818
Smoker	1.18	0.88–1.59	0.350	0.94	0.56–1.58	0.850

^1^ Reference: Class 2. ^2^ Single: includes separated, monk/nun. ^3^ Dependency in instrumental ADLs.

## Data Availability

The data presented in this study are available upon reasonable request from the corresponding author and approval of the funder.
